# Balancing DNA repair to prevent ageing and cancer

**DOI:** 10.1016/j.yexcr.2021.112679

**Published:** 2021-08-15

**Authors:** Eleanor Rachel Stead, Ivana Bjedov

**Affiliations:** a UCL Cancer Institute, Paul O'Gorman Building, University College London, 72 Huntley Street London, London WC1E 6DD, UK; b University College London, Department of Medical Physics and Biomedical Engineering, Malet Place Engineering Building, Gower Street, London WC1E 6BT, UK

**Keywords:** Ageing, Cancer, Longevity, Lifespan, Healthspan, DNA damage, DNA repair, Stem cells, Chromatin, Mutation, Epigenetic

## Abstract

DNA damage is a constant stressor to the cell. Persistent damage to the DNA over time results in an increased risk of mutation and an accumulation of mutations with age. Loss of efficient DNA damage repair can lead to accelerated ageing phenotypes or an increased cancer risk, and the trade-off between cancer susceptibility and longevity is often driven by the cell's response to DNA damage. High levels of mutations in DNA repair mutants often leads to excessive cell death and stem cell exhaustion which may promote premature ageing. Stem cells themselves have distinct characteristics that enable them to retain low mutation rates. However, when mutations do arise, stem cell clonal expansion can also contribute to age-related tissue dysfunction as well as heightened cancer risk. In this review, we will highlight increasing DNA damage and mutation accumulation as hallmarks common to both ageing and cancer. We will propose that anti-ageing interventions might be cancer preventative and discuss the mechanisms through which they may act.

## 1 Types and sources of DNA damage

**Fig. 1:**
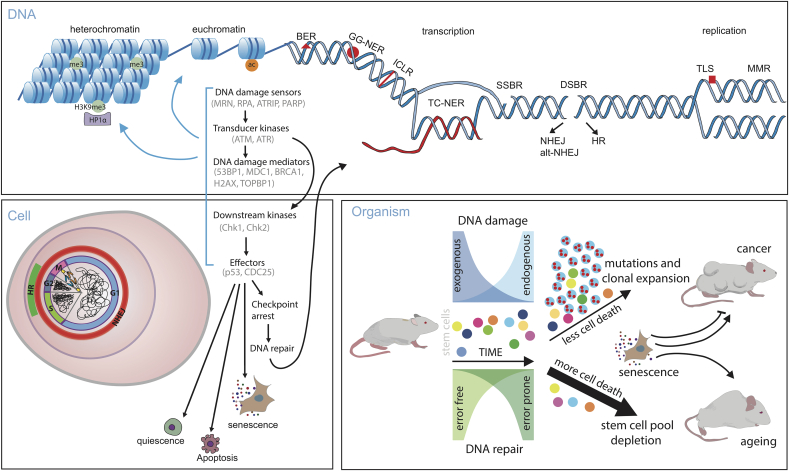
DNA damage and repair on DNA, cellular, and organismal level. DNA repair may differ if the damage is situated in the heterochromatin or euchromatin [5,6]. Heterochromatic regions have increased H3K9me3 mark, which is bound by HP1α (heterochromatin protein 1 alpha) [7]. Acetylation of histones in euchromatin increases chromatin availability [8]. Represented are different types of DNA repair, such as BER (base excision repair), GG-NER (global genomic nucleotide excision repair), ICLR (inter-strand crosslink repair), TC-NER (transcription coupled nucleotide excision repair), SSBR (single strand break repair), DSBR (double strand break repair), which can be repaired by HR (homologous recombination) or by either NHEJ (non-homologous end joining) or more mutagenic alt-NHEJ (alternative NHEJ) [9]. NHEJ is active throughout the cell cycle while HR is restricted to the late S and G2 phase. Replication errors are repaired by MMR (mismatch repair system) or are tolerated and bypassed by TLS (translesion synthesis repair). Transcription and replication make genome more vulnerable to damage and are associated with specialised types of repair. Upon DNA damage, damage sensors, such as PARP, mediate recruitment of transducer kinases ATM or ATR, whose activation leads to activation of DNA damage response to downstream proteins MDC1, BRCA1, 53BP1 and others. In presence of damage, ATM and ATR also activate Chk2 and Chk1, respectively [9]. CDC25 is one of the effector proteins that arrests cell cycle to allow damage repair [10]. p53 modifies transcription and thereby has a role in cell cycle arrest, apoptosis and senescence [11]. Mutations accumulate during ageing and are caused by different exposure to endogenous and exogenous factors, which produce DNA damage that can be repaired in error-free or error prone manner. Highly damaged cells are targeted for cell death, excess of which protects from cancer but depletes the stem cell pool and has pro-ageing effect [12,13]. Shown is clonal expansion of a mutated stem cell clone. If cell death is not induced in damaged and aberrant cells, then this increases chances clonal selection and expansion. Senescence is cancer protective but excess of prolonged senescence can promote both ageing and cancer via SASP (senescence associated secretory phenotype) [14].

DNA is subject to constant assault, an estimated 70,000 lesions occur per day for a typical human cell [1]. This damage can originate from endogenous sources, such as reactive oxygen species (ROS), enzyme action and replication errors. Depurination, depyrimidination, single strand breaks (SSBs), 8-oxoG and cytosine deamination are the most common forms of DNA damage that arise spontaneously [1]. ROS causes direct modification to DNA bases by oxidation and results in conversion of guanine to 8oxoG. Depurination arises from spontaneous chemical reactions, typically hydrolysis, which breaks the labile glycosidic bonds between the DNA base and the deoxyribose creating an abasic site. These sites can result in mutations due to misincorporation by DNA polymerase or translesion DNA synthesis (TLS) [2]. Spontaneous, hydrolytic reactions such as deamination, can also cause mutations, for instance cytosine deamination converts it to uracil, while deaminated 5-methylcytosine becomes thymine. This process results in mutation accumulation over time and the mutational signature of 5-methyl-cytosine deamination shows a strong positive correlation with the age of when cancer is diagnosed [3]. During DNA replication the frequency of these spontaneous reactions increases due to the exposure of more vulnerable ssDNA [4]. Therefore, in scenarios where ssDNA is exposed for prolonged periods such as replication or transcriptional stress, the likelihood of damage occurring increases (see Fig. 1).

Mitochondrial dysfunction and metabolic stress can result in increased production of reactive by-products such as ROS, lipid peroxides, oxidatively damaged proteins and aldehydes, of which lipid peroxidation may be particularly harmful due to its unique ability to propagate and amplify [15,16]. These reactive by-products, as well as endonuclease cleavage can cause endogenous SSBs and double strand breaks (DSBs) [16,17]. Another cause for SSBs and DSBs can be transcriptional stress due to abortive topoisomerase 2 action (TOP2CC cleavage complexes). The frequency of these events is increased in the presence of other lesions such as base cytotoxic modifications [18]. In addition, DNA damage lesions that are close in proximity, such as SSBs or closely spaced ongoing BER or NER on opposite strands, can further progress into DSB [19]. DSBs can also form when stalled replication forks collapse [20], further underlining how replication and transcriptional stress can contribute to endogenous DNA damage. The variety of causes for DSBs is important because, although this is the least frequent form of damage, DSBs are the most dangerous form of damage.

Multiple exogenous agents induce DNA damage, including UV light, ionising radiation, and chemical mutagens, such as polycyclic aromatic hydrocarbons present in tobacco smoke [[21], [22], [23]]. These agents cause chemical or physical modifications to the DNA often producing specific structures that are recognised by distinct repair enzymes. Interestingly, some cancer inducing chemicals do not seem to cause DNA mutations, but they induce selective constraint and expansion of existing clones instead, without generating specific mutation signatures [24]. Perhaps similarly, an “invisible” chemical or a metabolite, that does not leave fingerprints on DNA, could abet clonal expansion among hematopoietic stem cells (HSC) during ageing [25]. Another intriguing recent finding is that overexpression of some proteins, belonging to a variety of cellular functions, can also cause DNA damage and mutations [26].

Overall, DNA is continuously being damaged, often by unavoidable cellular metabolic processes. The challenge is to match a variety of different DNA lesions with the most adequate DNA repair enzymes and to coordinate the repair with other cellular activities.

## 2 How is DNA damage repaired? DNA damage repair pathways

Complex repair pathways have evolved to deal with the persistent problem of DNA damage [27]. They have been particularly well characterised in *E. coli*, yeast and mammals, and remarkable evolutionary conservation of repair enzymes have been observed between different organisms [28,9]. The repair pathway utilised depends on the type of damage incurred, the phase of the cell cycle and the availability of repair machinery. Below we describe DNA repair pathways, focusing mainly on mammalian systems.

Replication fidelity is preserved by the proofreading activity of polymerases [29], mismatch repair (MMR) [[30], [31], [32]], and regulation of the nucleotide pool's quality and quantity [33]. Lesions which block replicative DNA pol δ/*ε* can be bypassed using translational DNA synthesis (TLS) polymerases [[34], [35], [36]] or repaired by template switching (TS) [37,38]. Both TLS and TS, can be mutagenic and can be thought of as damage tolerance instead of repair.

Lesions that distort the DNA helix, such as bulky adducts or UV-light induced pyrimidine-dimers, are repaired by nucleotide-excision repair (NER). Lesions that do not alter the helical structure of DNA, such as 8-oxoG, uracil or an AP (apurinic/apyrimidinic) site, are repaired by base excision repair (BER). BER also acts upon SSBs. DSBs are repaired by homologous recombination (HR), non-homologous end joining (NHEJ) or alternative NHEJ (Alt-NHEJ), a microhomology-based pathway. Different repair pathways are predominant in different phases of the cell cycle, for example HR requires the presence of homologous or sister chromosomes and is therefore active during late S and G2 phase [39,40]. Pathway activity is determined by CDK activity which regulates the expression of the required repair factors. For example, CDK2 activity is required for the expression of CtIP which promotes end resection, a key step for the initiation of HR [41,42].

NHEJ is divided into classical NHEJ (c-NHEJ) and alternative NHEJ (Alt-NHEJ). HR is divided into gene conversion HR (GC-HR) and single strand annealing (SSA-HR) [42]. HR requires a plethora of repair enzymes and factors which are often shared with other repair pathways such as the Fanconi anaemia (FA) pathways [43] which repair interstrand cross links (ICLs) and template switching [44] which relies on the use of HR machinery e.g. BRCA2 and Rad51 [45].

The switch from NHEJ to HR is influenced by the complexity of the break site, by the availability of DNA repair components and the compaction of the chromatin [[46], [47], [48], [49]]. NHEJ is thought to be more erroneous than HR, however the cell repairs over 80% of DSBs by using NHEJ [46,[50], [51], [52]]. Even in G2 phase cells, NHEJ may be the first-choice pathway, as cells which lack DNA LigIV, a key component for NHEJ, not only exhibit a G1 phase repair defect but a G2 phase repair defect as well [46,48]. This may be due to the ease and speed of NHEJ. A subset of DSBs, around 15–20%, which cannot be repaired by NHEJ undergo end resection and repair by HR [46,53].

The choice between NHEJ and HR is also governed by levels of 53BP1. 53BP1 acts to restrain end resection whilst CtIP acts to promote it. However, 53BP1, along with its effector protein Shieldin [54,55] also determines whether HR is carried out by gene conversion (GC), typically thought of as an ‘error-free’ repair mechanism, or SSA which is a result of extensive end resection and is highly erroneous. In this way 53BP1 prevents excessive end resection and ensures the fidelity of HR [56]. Recent studies further indicate that HR can be mutagenic [40,57,58]. This is likely to be due to the involvement of translesion synthesis DNA polymerases such as Polζ [[59], [60], [61], [62]] and because the ssDNA generated in the repair process is more at risk of being damaged [63].

The repair of DNA must happen within the broader context of the nuclear landscape/architecture; therefore, chromatin dynamics and modifications are also key player in the repair. The DNA damage response invokes a large array of histone modifications, e.g. poly-ADP ribose (PAR) chains, γH2AX, H2A-Ubiquitination. These facilitate repair in multiple ways, for example, reducing local rate of transcription, opening up compacted chromatin regions or serving as recruitment or signalling platforms for repair enzymes/processes [56,[64], [65], [66], [67], [68]]. High levels of chromatin accessibility and transcription are associated with fewer base-pair substitutions perhaps due to more efficient MMR or TC-NER [[69], [70], [71]]. Mutation rate in euchromatic, early replicating regions of the DNA is reduced compared to late-replicating, heterochromatic regions [72]. This may be due to early regions having more time to detect and repair faults while late-replicating regions are often rich in repetitive sequences which are problematic for polymerases. Chromatin accessibly is not, however, a guarantee of more proficient repair, as despite the DNA being accessible in transcriptionally active regions, the presence of DNA-binding factors such as transcription factors can cause exclusion of repair proteins [73]. Transcriptionally active and accessible regions tend to accumulate genomic rearrangements [74] and mutational hot spots [75], including regulatory regions [76,77].

Heterochromatic DSBs are preferentially repaired by HR [48,49,78,79]. The co-localisation of γH2AX, 53BP1, and MDC1 is exclusive to areas of H3K9me3, a mark of condensed chromatin. This suggests the assembly of these factors is promoted in heterochromatic regions and may contribute to the preference of HR in heterochromatic regions [80]. Within heterochromatin, DSB repair may rely on a specific HR pathway that is dependent on ATM and involves Artemis, 53BP1, RNF168 and RNF8 [81]. Repair efficiency has also been correlated with the mobility of DSBs. Heterochromatic DSBs are often extruded to the periphery of the heterochromatic domain to undergo repair [5]. However, extrusion of DSBs also occurs for irreparable DSBs. They are pushed out to the nuclear periphery as a last resort to prevent interference with undamaged DNA [6]. Recently, heterochromatin, specifically H3K9me3 marks, have been associated with mechanosensing. Nuclear softening driven by loss of H3K9me3, protects the cell from DNA damage induced by mechanical stress [82].

The cell is equipped with a remarkable set of tools to repair DNA. However, it is essential that the least mutagenic repair complex gets priority access to its cognate lesion, and that these repair enzymes, capable of cutting, resecting, and ligating DNA, are firmly controlled to avoid mutations and chromosomal aberrations.

## 3 **How does the cell respond to DNA damage?** DNA damage detection, checkpoint arrest, and choice of cell fate

A key element in DNA repair is damage detection. Major detectors of single and double strand DNA breaks are PARP1 (poly(ADP-ribose) polymerase 1) and PARP2 (poly(ADP-ribose) polymerase 2) enzymes, which signal broken DNA by decorating adjacent histones with poly(ADP-ribose) chains, that at the same time relax chromatin and increase access of DNA repair proteins to the damage [66,83]. Often, PARP enzymes are aided by PARP complex accessory proteins, such as HPF1 (histone PARylation factor 1), which limits PARylation to serine residues, a typical post-translational modification of DNA repair proteins, rather than other residues such as glutamate and aspartate [[84], [85], [86], [87]]. PARylation is an early, brief event in DNA repair, which is terminated by removal of PAR chains by PARG and ARH3-mediated hydrolysis, once DDR factors are recruited to the lesion [66,83]. The importance of PARP enzymes in DNA repair is exploited in cancer therapy and PARP inhibitors have shown great success, specifically in HR-defective cancers [88,89]. A critical feature of PARP inhibitors is trapping PARP enzyme on broken DNA, which instead of initiating DNA repair generates further obstruction and damage and can lead to replication fork collapse. This creates an excess in the amount of substrates for recombination repair, which in HR-deficient cancer cells, such as BRCA1 mutated tumours, is limited to repair by lower-fidelity NHEJ, leading to chromosomal aberration such as radial chromosomes and selective death of tumour cells [88,89].

Another essential control of DNA damage response is brought about by phosphoinositide 3-kinase (PI3K)-related kinases: ATM, ATR and DNA-PKcs. These kinases are recruited to DNA breaks by their corresponding interacting proteins, for instance NSB1 from the Mre11/Rad50/NSB1 (MRN) complex recruits ATM double strand breaks. DSBs are also recognised by the Ku80 protein thereby aiding DNA-PKcs access to DNA damage. ATRIP, bound to RPA, is activating ATR kinase once replication forks are stalled [90]. These kinases provide a critical signalling cascade that orchestrates and activates a variety of DNA repair proteins that are specialised for given lesions [90].

The DNA repair process is helped by the cell cycle checkpoint arrest. The DNA damage response must be rapid and occurs before transition to the next phase of the cell cycle. Induction of checkpoint arrest relies on phosphorylation events such as ATR/ATM kinases acting to phosphorylate Chk1/2. Whereas maintenance of the checkpoint, whilst repair is occurring, relies on slower mechanisms involving transcription and expression of p53 and p21 [91].

Repair networks are brought back to homeostasis by the action of phosphatases, such as Wip1 and PP2A, to inactivate the DDR (DNA damage response). Depending on the level of DNA damage, several cycles or oscillations of effector proteins such as p53 [91] occur until the cell manages to repair the damage or a threshold is reached at which the cell commits to apoptosis, senescence or alternative cell death mechanisms due to prolonged activation of the DDR. The choice of cell fate, such as senescence, apoptosis, and quiescence, is governed by a combination of several factors, including the degree and type of damage, the activity of the p53-p21 axis, growth and survival signalling through PTEN-PI3K-AKT-mTOR and MAPK, the cell type, and the environment. The type and level of DNA damage, the efficiency of repair and the cellular response to this damage dictates whether an organism becomes more prone to cancer (by preserving mutated cell) or exhibits accelerated ageing phenotypes (by excessively eliminating cells with genomic aberations).

## 4 Somatic mutations are common characteristics of both cancer and ageing

Here we will present data showing how mutation accumulation is a common characteristic of both cancer and ageing [92,12], and some recent findings showing that non-tumorigenic normal tissue has surprisingly high mutation burden [93]. Cancer is a disease initiated and fuelled by genetic mutations, with multiple ‘hits’ required to malignantly transform a cell [93,94] and failure of DNA repair mechanisms may result in mutations [95]. In lung and skin cancers, mutation rates are dramatically increased by exposure to tobacco smoke and UV light, respectively [3,95,96]. Mutation rates in normal, somatic cells (B and T cells, fibroblasts, retinal and intestinal epithelium) is reported to be in the order of 2–10 mutations per cell division [97]. However, the incidence of cancer cannot be explained by this rate alone as the number of driver mutations generated would be insufficient to cause cancer. Instead, clonal expansion and hyper-mutation have been proposed to increase both the number of cells at risk and account for the discrepancy between mutation frequencies and cancer rates [98,99]. The predicted tissue-specific risk factor for cancer was proposed to be largely determined by stem cell endogenous replication error rates as opposed to exposure to exogenous factors [100,101], although there are other factors at play [102,103]. The exact number of driver mutations required to cause cancer is still unknown, and this may depend on the type of cancer and type of mutations acquired. Recent studies have shown that tobacco smoking, despite inducing a high mutational burden in the lung epithelial cells, leaves a population of quiescent cells which escape the high levels of DNA damage. These ‘protected’ cells go on to repopulate the lungs in those who stop smoking [104]. This highlights the cellular heterogeneity of mutation and how the selective process of regeneration can impact the mutational landscape of whole tissues over time.

Beyond cancer, accumulation of somatic mutations is thought to play a key role in ageing. Since mutations accumulate during ageing, this likely explains why ageing is the major risk factor for cancer [12,13]. The accumulation of somatic mutations in normal tissues is not well understood, they occur spontaneously throughout life and in a tissue-dependent manner. In the skin of the eyelid of normal, healthy persons, thousands of point mutations have been acquired by middle age and approximately 30% of cells have at least one driver mutation [105]. Daily exposure to sun light will have increased the number of mutations in the skin, however in the oesophagus, hundreds of clones are still present per square centimetre of tissue and these somatic mutations accumulate with age of the donor [106]. Recent single cell genome analysis of liver cells revealed the differentiated cells (hepatocytes) harbour higher levels of mutations accumulated with age compared to the adult stem cells [107], indicating certain cell populations, namely stem cells, are protected to an extent. This accumulation of mutations and clonal expansions in aged persons may contribute to the significant increase in cancer risk with age, from 2% risk at the age of 40, to a 50% risk by the age of 80 [93]. The accumulation of mutations and clonal expansion may lead to tissue dysfunction while changes in the tissue environment, such as inflammation, may drive further clonal selection and expansion [[108], [109], [110]]. While other unknown mechanisms which constrain clonal expansion may contribute to protection against cancer with age [111].

Overall, this common hallmark of cancer and ageing suggests that more crosstalk between these fields is urgently needed for better and faster understanding of the underlying causes of mutation accumulation.

## 5 Insights about cancer and ageing from DNA repair and growth signaling pathway mutants

Interesting insights about ageing and cancer could be gained by examining phenotypes of different DNA repair mutants, some of which are pro-ageing while others pro-cancer. In addition, in recent years there is evidence that down-regulation of growth pathway signalling can impact cancer and as well as ageing.

Several human disorders characterised by accelerated ageing are caused by deficiencies in DNA repair pathways. They often exhibit a high cancer incidence [112,113]. Examples include Werner and Bloom syndromes, both caused by mutations in the RecQ type helicases [114]. When modelled in yeast and mice, pro-ageing and pro-cancer phenotypes are observed as well as an increase in mutation frequency [[114], [115], [116], [117], [118]]. Pro-ageing and pro-cancer phenotypes are also seen in mice deficient in other key DNA damage repair proteins such as ATM [119] and p53 [120] suggesting that a functional DNA damage repair system is required for both cancer protection and longevity. Several studies in animal and cell models, in which the amount of DNA damage and mutations has been altered by deactivating or over-activing DNA repair genes, result in accelerated or decelerated ageing, respectively [12,13]. Overexpression of DNA repair genes in *Drosophila* including loki (Chk2), mei-41 (ATR) and WRN amongst others extend lifespan [[121], [122], [123], [124]]. It should be noted however that overexpression of DNA repair enzymes led to either longer or shorter lifespan, depending on target tissue, level of expression and sex of the animals tested, therefore more investigation is needed to understand how DNA repair can be enhanced [123]. Given that the DNA repair process requires that numerous signalling proteins and repair enzymes act in concert, it is challenging to enhance DNA repair by overexpressing a single enzyme. More promising, albeit more pleiotropic, would be to alter some of the upstream regulatory pathways. For instance, enhanced capacity for DNA repair is reported in the long-lived Ames and Snell dwarf mice, in which IIS is reduced [125,126]. Ames dwarf mice also exhibit delayed accumulation of spontaneous mutations as do mice which are subject to caloric restriction, a regime which reduces IIS/mTOR signalling and extends lifespan [127]. Furthermore, in Snell dwarf and growth hormone receptor knock-out (GHR-KO) mice the downregulation of TORC1 activity was linked to upregulation of several proteins involved in DNA repair [128]. These data suggest that the down-regulation of IIS/mTOR signalling may promote longevity through upregulation of DNA repair pathways resulting in reduced age-associated mutation accumulation. Reduced cancer incidence is observed in the long-lived GHR-KO, Ames dwarf and Snell dwarf mice [129,130] as well as in mice treated with rapamycin [131,132]. However, other long-lived models, such as S6K1−/− mice do not show any difference in tumour incidence compared with controls [133] but they do show reduced incidence of other age-related pathologies. In yeast deletion of the S6K homologue (Sch 9) reduces genomic instability with age [134] and further deletion of homologues for TOR and Ras combined with the Sch 9 deletion produces a four-fold extension in lifespan with reduced age-related mutational frequency and genomic instability [135]. These studies suggest there is a strong link between the lifespan extending mechanisms of reduced IIS/mTOR signalling and genomic stability in old age which may explain the reduced cancer incidence often observed in long-lived animal models. Long-lived mutants are often also healthier, nevertheless it is important for future potential translational approaches that health improvements and healthspan, or the period of good health of individual mutants is also carefully characterised as well as longevity [136].

Interestingly, mouse models of accelerated-ageing syndromes which exhibit high levels of DNA damage (NER deficiency) also show attenuated IGF-1 signalling [137]. Initially this was puzzling because if IGF-1 signalling was reduced, why was lifespan not extended in these mice? It is now thought that cells respond to DNA damage by decreasing IGF-1 signalling to re-direct resources from growth to maintenance, therefore reducing IGF-1 signalling may act to enhance DNA damage repair in normal, healthy cells. For progeria-like syndromes however or DNA repair mutants the re-direction of resources is not enough, the high levels of DNA damage due to the deficiency in DNA repair ultimately leads to cell death, stem-cell functional decline and accelerated ageing [113,137,138]. However, when placing these NER deficient mice on caloric restriction, further downregulating IGF-1 signalling, this significantly extended their lifespan, health-span and increased genomic stability [139].

Comparative studies of several mammalian species have revealed positive correlations between DNA repair efficiency and lifespan [[140], [141], [142], [143]]. The long-lived naked mole rat upregulates several genes involved in DNA damage repair resulting in more efficient base-excision repair (BER), mismatch repair (MMR), double strand break (DSB) repair and upregulation of the tumour suppressor gene, TP53, promoting cancer resistance [[144], [145], [146], [147], [148], [149]]. Other mechanisms proposed for the naked mole rats cancer resistance include secretion of high molecular-mass hyaluronan which renders cells hypersensitive to contact inhibition, and these cells stop proliferating upon only a few cell-cell contacts [150].

p53 is a key player in the DNA damage response. In mice constant over-activation of p53 (p53+/mut) results in protection from cancer, likely due to heightened cell death but at the expense of a shorter lifespan [151], suggesting there is a trade-off between longevity and cancer protection, and that cancer protection can only be achieved at the expense of shorter lifespan. However, cancer protection and a normal lifespan is seen in super-p53 mice, which has an extra copy of p53 driven by the native promoter providing enhanced DNA repair capacity but only when required [152]. Interestingly, it is only when super-p53 mice have an additional copy of the tumour suppressor p19Arf that lifespan extension is achieved alongside cancer protection demonstrating that an anti-ageing and anti-cancer phenotype can be achieved, despite multiple examples of this trade-off [153]. The trade-off between pro-ageing but cancer protective mechanisms and anti-ageing yet pro-cancer phenotypes is often observed [[13], [154]] and may be explained by differing responses to DNA damage. A response which leads to excessive apoptosis or senescence provides protection against mutation and therefore cancer, but at the cost of a pro-ageing phenotype due to stem cell pool depletion or accumulation of senescent cells. It should be noted that evidence for stem cell pool depletion is primarily observed in DNA repair mutant animals or in normal cells and animals that have been exposed to stressors or exogenous agents. The evidence regarding stem cells being depleted under the normal ageing process is scarce [[155], [156], [157]], and it is more likely that during normal ageing stem cell functional decline occurs [158].

On the other hand, a lack of cell death following DNA damage may promote longevity, but favours the accumulation of mutations and build-up of pre-malignant cells, resulting in elevated cancer risk. This trade-off phenomenon is exemplified by comparing Cockayne Syndrome and Xeroderma Pigmentosum (XP) both of which are a result of deficiencies in NER. Cockayne Syndrome is due to mutations in the CSA (Ercc8) or CSB (Ercc6) genes involved in the first steps of transcription-coupled repair (TC-NER). When cells with TC-NER deficiency sustain DNA damage they die due to transcriptional stress. This results in an accelerated ageing phenotype because the stem cell pool is depleted, but no cancer arises as the damaged cells are eliminated before they accrue mutations. In Xeroderma pigmentosum, cells are deficient in global-genome nucleotide excision repair (GG-NER) due to mutation of the XPC gene. TC-NER is still functional in these patients and promotes cell survival which delays premature ageing. However, due to the lack of GG-NER, lesions occurring in the non-transcribed genomic regions or in the template strand of active regions frequently result in mutations during replication. Therefore cancer incidence is high in XP patients [159].

In summary, defects in DNA damage repair can result in an accumulation of mutations or increased cell death, promoting cancer or ageing, respectively. These phenotypes lie at two ends of the spectrum with several possible intermediary phenotypes. In the context of translational medicine, the most interesting and relevant mutants are those displaying both cancer resistance and delayed ageing, for example the previously mentioned super-p53/Arf mice [153]. Another example is the long-lived *C. elegans daf-2* (insulin receptor) mutant which shows resistance to lethal germ-line tumours caused by *gld-1* mutation [160]. The mechanism behind such resistance is thought to be due to increased apoptosis, in which the *daf-2* mutant background imposes a metabolic strain on the organism which results in selective apoptosis of the heavily, metabolically-demanding tumour cells but the surrounding normal tissue is not affected.

Growing evidence indicates cellular senescence occurring with age is a result of DNA damage accumulation [[161], [162], [163]]. Accumulation of senescent cells often results in an adverse senescence associated secretory phenotype (SASP) and can create a pro-tumorigenic environment [14]. The clearance of senescent cells extends lifespan and delays the onset of cancer [[164], [165], [166], [167], [168], [169], [170]].

During normal ageing the function and efficiency of several DNA repair pathways are thought to decline with age [171] including the p53 response [172]. A lack of efficient DNA damage repair combined with reduced apoptosis, due to an inefficient p53 response, may contribute to mutation accumulation with age. Overall, these studies demonstrate a functional DNA damage response is required to preserve genomic integrity which is essential for both longevity and cancer protection [173]. IIS/mTOR signalling regulates ageing and may contribute to some aspects of DNA damage repair and genomic stability. However, whilst it is expected that protection from DNA mutations will reduce cancer incidence, a careful balance is required between tumour suppression and maintenance of functional stem cell pool to ensure a long-life.

## 6 Stem cells in ageing and cancer

Stem cells play a critical part in renewing our tissues, but repeated cell divisions makes stem cells vulnerable to both transformation and cell death, depending on the type of mutations they accumulate [25]. Mutant clonal expansion and selection of stem cells occurs within the intestine and hematopoietic system with age [[174], [175], [176], [177], [178]]. In the intestine of humans and mice, this leads to clonal dominance of single ISCs within crypts [[179], [180], [181], [182]]. Similarly, in human skin, there is also evidence of clonal dominance and prevalence of clones bearing mutations in NOTCH1, NOTCH2, TP53 and FAT1 [157]. Intriguingly, NOTCH1's fitness advantage is restricted to the normal oesophageal epithelium because this mutation is not overrepresented in oesophageal cancer [106,183].

Most evidence for the role of ISCs in ageing comes from the fruit fly, *Drosophila*. ISCs are regularly interspersed throughout the *Drosophila* gut, unlike mammalian ISCs which are located in crypts. Damage to the fly gut stimulates ISC proliferation to replace dead or dying cells [[184], [185], [186], [187], [188]]. At a young age this is a transient effect, and the stem cells return to quiescence but in aged guts ISC over-proliferation and an increased mis-differentiation is observed [184,189,190]. Preserving proliferative homeostasis in the *Drosophila* gut extends lifespan and reduces the incidence of hyperplasia with age [187,191,192] highlighting the importance of ISC quiescence in maintaining tissue integrity with age. These studies did not report directly on ISC genomic integrity, but DNA damage and somatic mutations do accumulate with age in *Drosophila* [[193], [194], [195]]. Genomic aberrations arising in the ISCs can drive gut neoplasia and dysplasia [25,196,197]. Therefore *Drosophila*, like humans, also exhibits an increased cancer risk with age [198]. In the crypts of mammalian intestines, the LGR5^+^ ISCs are highly proliferative [199], therefore unlike in *Drosophila*, quiescence is not important to maintain tissue integrity with age. Instead Wnt signalling appears to be the dominant factor regulating survival and regenerative capacity of ISCs in response to both DNA damage and ageing [[200], [201], [202], [203]].

Replication stress is a key source of endogenous DNA damage contributing to both ageing and cancer progression, particularly when stem cells are affected. Ageing Haematopoietic Stem Cells (HSCs) are particularly vulnerable to replication stress, which has been attributed to reduced levels of mini-chromosome maintenance (MCM) helicase components [158,204,205]. The old quiescent HSCs have the DNA damage marker γH2AX primarily concentrated in the nucleolus, which houses the rDNA genes required for ribosome biogenesis. rDNA genes have several features, such as multiple repeat clusters and high transcription rates, that make them particularly vulnerable to DNA damage [206]. Dephosphorylation of γH2AX seems to be hampered in the old quiescent HSCs because of cytoplasmic mislocalisation of PP4c phosphatase, all of which may lead to a decrease in ribosomal biogenesis and therefore limits the functionality of aged HSCs and their ability to regenerate the blood-cell lineage [158,207]*.* Mutant HSC clones accumulate with age and contribute significantly to the age-related risk of leukaemia in humans [177,178,208,209].

HSCs enter quiescence when not actively required, however in this state they attenuate their DNA repair responses resulting in an accumulation DNA damage with age. It is only upon re-entry into the cell cycle that DNA repair occurs in these aged HSCs [210]. Whilst cycling, cells are more likely to experience mutations arising from replication errors [211], yet may benefit from HR of DSBs during the S and G2 phases. Quiescent cells, on the other hand, may rely on NHEJ, but evidence shows a preference for classical NHEJ and active suppression of the more erroneous alt-NHEJ [212] indicating an attempt to keep mutation rates low.

Replication stress can shorten lifespan in mice. Loss of MCM2 promotes premature ageing [213] consistent with observations in murine HSCs [158,207]. ATR deficient mice exhibit dramatically reduced regenerative capacity and accelerated ageing [204,205] which is reflected in the human disease, Seckel syndrome [214]. Replication stress is also observed in another accelerated ageing syndrome, Ruijs-Aalfs syndrome [215]. Here, loss of *Spartan* results in destabilized replication forks that is further aggravated by a lack of translesion synthesis [[216], [217], [218]].

The attrition of functional HSCs with age may underlie common age-related dysfunctions such as a poor immune system/response and poor wound healing/regenerative capacity. However*,* in *C. elegans* the loss of MCM2 actually extends lifespan [219] and in long-lived *daf-2* mutants’, MCM2 expression is decreased compared to wild-types [220]. The differences between these studies on the relationship between MCM2 levels and ageing may be explained by the fact that *C. elegans* is largely a post mitotic organism, meaning they do not suffer from high levels of replication stress except in the germline. Alternatively, deficiency in one repair pathway may perhaps cause compensatory upregulation of a different repair pathway, leading to genome protection.

In summary, stem cells can accumulate DNA damage and mutations, leading to clonal selection and expansion, with age in a tissue dependent manner. Although stem cells may possess a level of inherent protection against mutations [104,107], on a population level, this may be contributed to by a low threshold for apoptosis due to increased expression of pro-apoptotic proteins as well as mitochondrial priming [221].

## 7 Chromatin status in ageing and cancer

The role of epigenetic modification in facilitating access to DNA damage and in having a more direct role in DNA repair, has been increasingly recognised [[222], [223], [224]]. Here we will describe the intricate relationship between epigenome and DNA repair which ultimately affects ageing and cancer.

Several changes to histone expression and methylation are observed upon ageing which may affect chromatin structure [225]. Ageing organisms and senescent cells exhibit reduced levels of repressive heterochromatic marks including H3K9me3, H3K27me3, and H4K20me3 [[226], [227], [228], [229]] and an overall global loss and redistribution heterochromatin is a characteristic feature of ageing [230,231].

Levels of heterochromatin protein 1 (HP1α) are diminished in aged human cells and prematurely aged cells [232,233]. The level of H3K9me3 are reduced in both aged fibroblasts and fibroblasts isolated from patients with HGPS (Hutchinson-Gilford progeria syndrome), a premature ageing syndrome [234]. Cellular models of the accelerated ageing syndrome, Werner's, also report a global loss of H3K9me3 and an interaction with HP1α [235]. In *C. elegans,* lifespan is extended by inhibition of H3K27 demethylases but this lifespan extension could be mediated by the associated changes in IIS observed in these worms [236]. In *Drosophila*, a lack of functional HP1 reduces lifespan and overexpression of HP1 improves longevity [237] suggesting that increases heterochromatin do promote longevity in *Drosophila*. These studies suggest disorganisation and loss of heterochromatin promote ageing and maintenance of heterochromatin with age increases longevity.

Links between epigenetic modifications and lifespan were first highlighted in studies of yeast in which Sir2, an NAD + -dependent histone deacetylase, was overexpressed [238,239]. Additional evidence for the role of sirtuins in ageing comes from work on SIRT6. SIRT6 deficiency in mice results in premature ageing [240]. Overexpression of SIRT6 reduces genomic instability [241], increases DSB repair efficiency by both the HR and NHEJ pathways and extends lifespan [149,242]. Of the two activities associated with SIRT6, mono-ADP-ribosyl transferase and histone acetylase, the former is proposed to increase DSB repair efficiency via PARP1 activation [243]. The authors highlight how longevity across several species correlates with DSB repair efficiency and not with efficiency of other repair pathways such as NER. Instead NER efficiency shows correlation or coevolution with the level of sun exposure per species [149]. The deacetylase activity could still affect DNA damage via the level of chromatin compaction. A lack of deacetylase activity would relax the chromatin and potentially increase exposure to DNA damaging agents [244]. Since the histone deacetylase activity of sirtuns depends on NAD + levels, as does the activity of multiple DNA damage repair enzymes, including PARPs, it has been suggested that maintaining NAD + levels, which are known to decline with age, may protect the DNA and have anti-ageing effects [[245], [246], [247]].

Another epigenetic modification, DNA methylation, is also closely linked to ageing [248]. This has been exemplified through the recent advent of epigenetic ageing clocks used to predict biological age from the DNA methylome [[249], [250], [251]]. Well-known anti-aging interventions, such as dietary restriction, also have measurable effects on the epigenome [252]. Chromatin remodellers play key roles in DNA repair, genome stability and in preventing tumorigenesis [222,253,254]. Chromatin genes that are often mutated in cancer, for example H3.3 which is linked to paediatric glioblastoma [255,256], also have roles in lifespan regulation [257,258].

Overall, links between epigenetic modifications and ageing are complex due to the gene regulation that accompanies changes in chromatin packaging. However, it is clear that the regulation of the chromatin affects both DNA repair efficiency and subsequent mutation rate both of which contribute to ageing and cancer. Most studies indicate that maintenance of heterochromatin throughout ageing and efficient DSB repair promote longevity, yet how heterochromatin affects cancer risk/rate is not yet clear.

## 8 Summary and future prospects

Maintaining genome integrity is key for longevity and reduced cancer risk and we argue here that interventions that lower mutations are expected to improve ageing and delay cancer. It should be noted that in a controlled and limited way mutations can be beneficial. For instance, normal functioning of the adaptive immune system and somatic hypermutation process depend on mutations introduced by AID (activation-induced cytidine deaminase), which enables production of antibodies with greater antigen affinity [259]. In single cell organisms, such as bacteria, mutations enable survival in presence of antibiotics [260], and mutation frequency is sometimes adjusted depending on stressful conditions in the environment [261]. Despite mutations being essential for evolution, they are mostly detrimental, and repair of DNA damage must therefore occur effectively to prevent both mutations and excessive cell death, which would otherwise lead to increased cancer risk or accelerated ageing, respectively. The high mutational burden of normal, somatic cells was surprising [[105], [106]], as is the discovery that particular populations of stem cells may be protected from DNA damage and mutation [104,107]. Both studies highlight how clonal selection and expansion are key factors in understanding cancer risk and ageing and are of interest for potential therapeutic interventions. Stem cells are key in the balance between cancer and ageing, it will be interesting to see whether alterations to chromatin status or reductions in replication and transcription stresses are present in ‘protected’ stem cells [262] and how this can be used for improvement of health and disease prevention. For understanding of high mutational burden in normal cells it will be important to clarify which endogenous and exogenous molecules are causing mutations [2] and some indication can be provided by mutational signatures [23,93,111,263]. Many commonly used laboratory chemicals that damage DNA, and which helped uncover details about DNA repair, are not necessarily chemicals that our cells most commonly encounter and more work on mutation causing agents in humans are needed [16]. Another future challenge would be to develop treatments that could boost error-free DNA repair. However, this is challenging because most DNA repair enzymes work in complexes and overexpression of one of them will not result in balanced and improved DNA repair. Expanding our knowledge around the enhancement of select aspects of DNA repair capacity and the mechanisms which can confer protection against DNA damage infliction will be critical to simultaneously promote longevity and reduce cancer risk.

## Credit author statement

Eleanor Rachel Stead: Conceptualization, Writing – original draft, Writing – review & editing, Visualization, Project administration, Funding acquisition, Ivana Bjedov: Conceptualization, Writing – original draft, Writing – review & editing, Visualization, Project administration, Funding acquisition.
